# Adipose tissue derived stromal vascular fraction as an adjuvant therapy in stroke rehabilitation: Case reports

**DOI:** 10.1097/MD.0000000000021846

**Published:** 2020-08-21

**Authors:** Hoon-Bum Lee, Si-Woon Park, Il-Kwon Kim, Jae-Hyung Kim, Doo Young Kim, Ki-Chul Hwang

**Affiliations:** a Department of Plastic and Reconstructive Surgery; b Department of Rehabilitation Medicine; c Cell Therapy Center; d Institute for Bio-Medical Convergence, Catholic Kwandong University International St. Mary's Hospital, Incheon, South Korea.

**Keywords:** case report, hemiplegia, mesenchymal stromal cells, rehabilitation, stroke

## Abstract

**Introduction:**

Stroke often causes residual hemiparesis, and upper extremity motor impairment is usually more disabling than lower extremity in those who are suffering from post-stroke hemiparesis. Cell therapy is one of the promising therapies to reduce post-stroke disability.

**Patient concerns:**

Three male participants were included in the study to investigate the feasibility and tolerability of autologous adipose tissue derived stromal vascular fraction.

**Diagnosis:**

All participants had hemiparesis after 1st-ever stroke longer than 6 months previously.

**Interventions:**

Under general anesthesia, liposuction of abdominal subcutaneous fat was performed. Stromal vascular fraction freshly isolated from the adipose tissue extract was injected into the muscles of paretic upper extremity. All participants received inpatient stroke rehabilitation consisted of physical and occupational therapy more than 3 hours a day for 2 months or more.

**Outcomes:**

The whole procedure did not produce any significant adverse event in all participants. Adipose tissue extracts yielded sufficient stromal cells. One participant showed clinically important change in upper extremity Fugl–Meyer assessment after the injection and it lasted up to 6 months. Functional magnetic resonance imaging showed concomitant increase in ipsilesional cortical activity. The other 2 participants did not show remarkable changes.

**Lessons:**

Intramuscular injection of autologous adipose tissue derived stromal vascular fraction seems to be a safe and tolerable procedure in subjects with chronic stroke, and its utility in rehabilitation needs further investigation.

## 1 Introduction

Stroke is a leading cause of acquired disability worldwide and the most common type of motor impairment after stroke is hemiparesis. For those who are suffering from post-stroke hemiparesis, upper extremity motor impairment is often much more disabling than lower extremity. It has been known that recovery from severe arm paresis is very limited.^[1]^ Efforts have been made to minimize disability following post-stroke hemiparesis.^[2,3]^ Studies have revealed that repetitive task-oriented rehabilitation resulted in functional recovery after stroke by inducing cortical reorganization.^[4,5]^ To maximize functional recovery, intensive rehabilitation as far as patients tolerate is recommended.^[6]^

Beside many rehabilitation therapies being developed, stem cell therapy is one of the promising therapies to reduce post-stroke disability. Stromal vascular fraction (SVF) extracted from adipose tissue has been known to contain rich of mesenchymal stem cells.^[7]^ Freshly isolated adipose stromal cells can be easily injected to human for clinical purposes.^[8]^ These cells release neurotrophic factors that can enhance brain plasticity.^[9]^ Previous animal study revealed stem cells injected into skeletal muscles affected remote organ by paracrine mechanism.^[10]^ In this study, it was assumed that stromal cells injected into muscles may act as a source of paracrine effect to enhance neuroplasticity in subjects undergoing rehabilitation. The purpose of the study is to investigate the feasibility and tolerability of autologous adipose tissue derived SVF in subjects with stroke, and its clinical utility as an adjuvant therapy for rehabilitation.

## 2 Methods

### 2.1 Participants

Three male participants with hemiparesis who had 1st-ever stroke at cerebral hemisphere longer than 6 months previously were included in the study. Participants’ characteristics are summarized in Table 1. This study was approved by the Institutional Review Board and written informed consent was obtained from all participants before enrolment for publication of this case report and accompanying images (IRB approval number: IS16BISE0010).

**Table 1:**
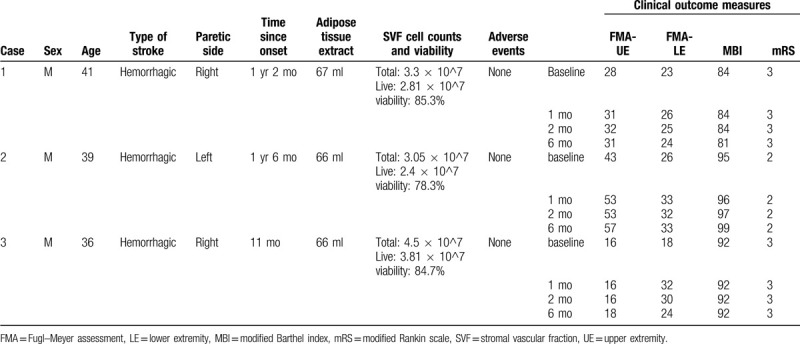
Participants’ characteristics, results of adipose tissue extraction and stromal cell isolation, and clinical outcome measures.

### 2.2 Intervention

Under general anesthesia, liposuction of abdominal subcutaneous fat was performed. Tumescent fluid made with normal saline 1000 mL, 2% lidocaine and 1 mL of 1:1000 epinephrine, was infiltrated into subcutaneous fat layers of abdominal wall, and then liposculpture was performed.

Extracted adipose tissue was centrifuged at 3000 rpm for 5 minutes to remove water, tumescent solution and oil. The 50 mL of fat tissue was digested with 0.075% collagenase type 1 at 37°C for 30 minutes under gentle agitation. After the digested tissue was filtered through 75 μm strainer to remove residual tissue, the cell suspension was centrifuged and washed with phosphate buffered saline 3 times at 3000 rpm for 3 minutes. Total and live cell counts were performed using the nucleocounter NC- 200TM automated cell counter (chemometec, Denmark).

SVF freshly isolated from the adipose tissue extract were injected into the muscles of paretic upper extremity.

All participants received inpatient stroke rehabilitation consisted of physical and occupational therapy more than 3 hours a day for 2 months or more.

The SVF injection was given after baseline assessment in 2 participants. For the third participant, the SVF injection was given 1 month later in order to compare the effect of rehabilitation for a month before and after the injection.

In all participants, liposuction procedure did not produce any significant adverse events, and the adipose tissue extracts yielded sufficient stromal cells (Table 1).

### 2.3 Outcome measurement

Primary outcome measure was Fugl–Meyer Assessment (FMA) for upper extremity. Its scores range from 0 to 66. Increase of 4.25 to 7.25 points or more in FMA score can be interpreted as clinically important difference.^[11]^ Secondary outcome measures included FMA for lower extremity (scores range from 0 to 34), modified Barthel index (MBI), and modified Rankin scale (mRS) for overall disability measure. For neurophysiologic measure, functional magnetic resonance imaging (fMRI) was taken while subjects perform repetitions of wrist or hand movement. All assessments were performed at baseline, 1 and 2 months after inclusion and 6 months after the SVF injection.

## 3 Results

### 3.1 Case 1

A 41-year-old male participant had intracerebral hemorrhage in the left basal ganglia a year and 2 months previously and was treated conservatively. He has been suffering from right hemiplegia since then. He had been diagnosed as having hypertension, and his blood pressure was well controlled at enrollment. He denied any other medical comorbidity or medications that may hinder surgery under general anesthesia. He had moderate arm paresis and the baseline FMA score for upper extremity was 28. He was able to walk under supervision, and the baseline FMA score for lower extremity was 23. The baseline MBI score was 84, and mRS was 3 (moderate disability).

**Figure 1:**
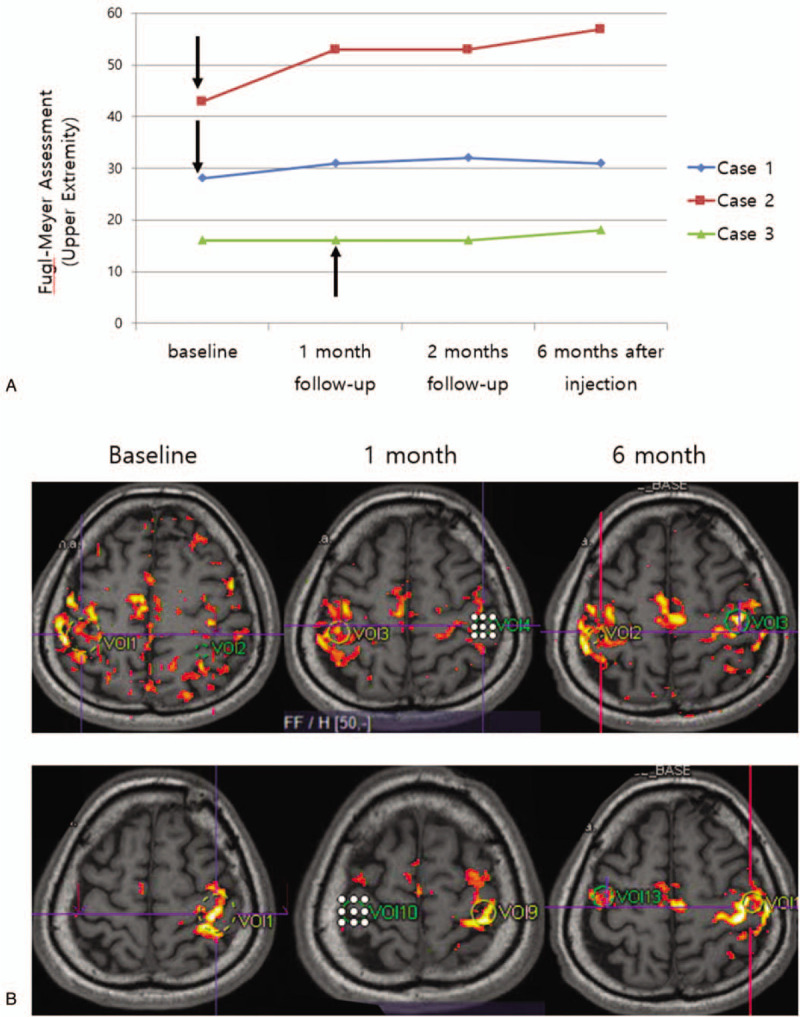
(A) Fugl–Meyer assessment for upper extremity of each cases before and after procedure. Arrows indicate time of injection. (B) Functional MRI changes over time in Case 2. Upper row is for moving the affected hand, and lower row is for moving the unaffected hand. See text for explanation. MRI = magnetic resonance imaging.

At 1 month after the injection, the FMA score for upper extremity was increased to 31, which is 3 points increase from the baseline, but he did not report any remarkable improvement. The FMA score for upper extremity at 2 and 6 months after the injection was 32 and 31, respectively (Fig. 1). Though the increment in FMA was maintained up to 6 months, he did not feel subjective improvement. The FMA score for lower extremity at 1, 2, and 6 months after the injection was 26, 25, and 24, respectively. The other outcome measures remained unchanged.

fMRI showed bilateral activation mostly in pre- and postcentral gyri when moving the affected hand, and no remarkable difference was noted after the procedure.

### 3.2 Case 2

A 39-year-old male participant had intracerebral hemorrhage in the right basal ganglia a year and 6 months previously and had surgical treatment to remove hematoma. He has been suffering from left hemiparesis since then. He had been diagnosed as having hypertension, and his blood pressure was well controlled at enrollment. He had been a hepatitis B carrier, but his liver function tests were within normal limits and he was not contraindicated for surgery under general anesthesia. He had mild arm paresis and the baseline FMA score for upper extremity was 43. He was able to walk under supervision, and the baseline FMA score for lower extremity was 26. The baseline MBI score was 95, and mRS was 2 (slight disability).

At 1 month after the injection, the FMA score for upper extremity was increased to 53, which is 10 points increase from the baseline, and he reported that his left thumb and index finger movement was improved. The FMA score was maintained at 2 months after the injection, but at 6 months it increased 4 more points up to 57 (Fig. 1). He reported improvements not only in hand function but also in sensory function and shoulder strength. The FMA score for lower extremity was increased as well, which was 33, 32, and 33 at 1, 2, and 6 months after the injection, respectively. He became able to walk independently. The MBI score was increased 1 to 4 points from baseline, and mRS remained unchanged.

Baseline fMRI showed bilateral activation in pre- and postcentral gyri, precuneus and middle frontal gyrus when moving the affected hand. After intervention, the active area during moving affected hand was more concentrated to ipsilesional pre- and postcentral gyri (Fig. 1).

### 3.3 Case 3

A 36-year-old male participant had intracerebral hemorrhage in the left basal ganglia 11 months previously and had navigation guided hematoma aspiration. He has been suffering from right hemiplegia since then. He had been diagnosed as having hypertension, and his blood pressure was well controlled at enrollment. He denied any other medical comorbidity or medications that may hinder surgery under general anesthesia. He had severe arm paresis and the baseline FMA score for upper extremity was 16. He was able to walk independently, and the baseline FMA score for lower extremity was 18. The baseline MBI score was 92, and mRS was 3 (moderate disability).

After a month of inpatient rehabilitation, upper extremity FMA score was unchanged but lower extremity score increased to 32. After the injection, he reported that his right arm function was somewhat improved. However, the FMA score for upper extremity was still 16 at 1 month after the injection, and it increased only 2 points at 6 months (Fig. 1). The FMA score for lower extremity at 1 and 6 months after the injection was 30 and 24, respectively. The other outcome measures remained unchanged.

fMRI showed bilateral activation mostly in pre- and postcentral gyri when moving the affected hand, and no remarkable difference was noted after the procedure.

All the results are summarized in Table 1.

## 4 Discussion

There have been trials of stem cell therapy to reduce post-stroke disability. Many researchers used bone marrow-derived mesenchymal stem cells,^[12]^ and adipose tissue-derived mesenchymal stem cell therapy was also tried recently.^[13]^ The mechanism of action of stem cells, as early studies indicated, is not likely to be cell replacement but its paracrine effect.^[12]^ While most stem cell trials targeted acute stroke, some researchers applied mesenchymal stem cells to subjects with chronic stroke.^[14,15]^ Stem cell therapy in acute stage stroke usually aims for neuroprotection, whereas neuroreparative effect of stem cell becomes main focus in later stage.^[16]^ In order to optimize its regenerative effect, integration of rehabilitation with stem cell therapy, so-called “regenerative rehabilitation,” has been advocated.^[17–19]^ When applying stem cell therapy to stroke population, a recent cohort study suggested that post-stroke upper extremity impairment should be the main target of therapy.^[20]^

Among the cases in this study, only 1 case with mild arm impairment gained meaningful benefit from rehabilitation combined with the SVF injection. Though its efficacy is neither confirmed by this single case nor strong enough to help those with severe paresis, there is possibility that SVF enhances rehabilitation effectiveness by neurotrophic paracrine effect. Considering the risks of surgery in stroke patients, manufactured extracellular vesicles may be safer option in future cell therapy.^[21]^ It should be stressed that any kinds of regenerative therapy have to be integrated with rehabilitation to get therapeutic benefit.

In conclusion, intramuscular injection of autologous adipose tissue derived SVF seems to be a safe and tolerable procedure in subjects with chronic stroke, and possibly has a potential to enhance the effect of rehabilitation in selected cases. For its clinical application, risks of surgery under general anesthesia as well as potential benefits of stromal cells to enhance neuroplasticity should be considered. Further investigation is requested.

## Author contributions

**Conceptualization:** Lee HB, Park SW.

**Data curation & formal analysis:** Park SW, Kim DY.

**Investigation:** Lee HB, Park SW, Kim IK, Kim JH.

**Methodology:** Lee HB, Park SW.

**Resources:** Hwang KC.

**Writing – original draft:** Park SW.

**Writing – review & editing:** Lee HB, Kim IK, Kim DY.
